# Lupus retinopathy: epidemiology and risk factors

**DOI:** 10.5935/0004-2749.20210076

**Published:** 2021

**Authors:** Leonardo Gomes Bortoloti de Azevedo, Ana Luiza Biancardi, Renata Alves Silva, Nycholas da Costa Tavares, Mirhelen Mendes de Abreu, Blanca Elena Rios Gomes Bica, Haroldo Vieira de Moraes Jr

**Affiliations:** 1 Ophthalmology Department, Universidade Federal do Rio de Janeiro, Rio de Janeiro, RJ, Brazil; 2 Rheumatology Department, Universidade Federal do Rio de Janeiro, Rio de Janeiro, RJ, Brazil

**Keywords:** Lupus erythematosus, systemic/epidemiology, Retinal diseases, Risk factors, Lúpus eritematoso sistêmico/epidemiologia, Doenças retinianas, Fatores de risco

## Abstract

Lupus retinopathy is a clinical manifestation of systemic lupus erythematosus in the visual system. It is generally asymptomatic; however, it can become a threatening condition. It is closely associated with the inflammatory activity and higher mortality of systemic lupus erythematosus. Lupus retinopathy has several different clinical presentations, such as lupus microangiopathy, vascular occlusion, vasculitis, hypertensive retinopathy associated with lupus nephritis, and autoimmune retinopathy. Although the prevalence and associated factors of lupus retinopathy have been well defined in some parts of the world, there are no data from Latin America, including Brazil. As lupus retinopathy is generally asymptomatic, without a routine fundoscopy, it has been probably underestimated. This review is intended to discuss the epidemiology and risk factors of lupus retinopathy.

## Abstract (pt)

A retinopatia lúpica é uma manifestação clínica do lúpus eritematoso sistêmico no sistema visual. Geralmente assintomática, porém pode ser uma condição ameaçadora à visão. Está intimamente associada à atividade inflamatória do lúpus eritematoso sistêmico e ao aumento da mortalidade. A retinopatia lúpica tem diversas apresentações clínicas, como a microangiopatia lúpica, oclusão vascular, vasculite, retinopatia hipertensiva associada à nefrite lúpica e retinopatia autoimune. A prevalência e os fatores associados à retinopatia lúpica estão bem definidos em algumas partes do mundo. No entanto, esses dados são pouco conhecidos na América Latina, incluindo o Brasil. Como a retinopatia lúpica é geralmente assintomática, sem a fundoscopia de rotina, provavelmente esta é subestimada. O objetivo desta revisão é discutir a epidemiologia e fatores de risco para retinopatia lúpica.

## INTRODUCTION

Lupus retinopathy (LR) is an ophthalmic presentation of systemic lupus erythematosus (SLE)^(1-13)^ and can be a threatening vision disease. The pathophysiology of LR is believed to be primarily related to the deposition of immune complexes in the retinal microvasculature, leading to vascular occlusions, microinfarcts, and retinal vasculitis^(14-19)^.

LR has a broad spectrum of manifestations, ranging from asymptomatic cases to severe visual loss^(1)^. In general, LR is bilateral, although it may be unilateral or asymmetric^(2)^. It is probably associated with disease activity, which can be measured using the SLE disease activity index (SLEDAI)^(4)^. Its criteria are related to clinical manifestations and laboratory results of SLE. The number of criteria found at the time of clinical appointment defines the score, which ranges from 0 to 105 points^(20)^. Higher scores are associated with severe SLE activity ^(21-23)^.

LR is most commonly found in hospitalized patients compared with well-controlled patients and outpatients. A prospective study conducted by Stafford-Brady et al. reported that 88% of patients with LR had active systemic disease and 73% had active central nervous system (CNS) involvement^(24-26)^. Although LR generally has good visual prognosis, it is a poor indicator for survival marker^(13)^.

### Clinical presentation of LR

LR has several different presentations. It can be observed as lupus microangiopathy, vascular occlusion, vasculitis, hypertensive retinopathy associated with lupus nephritis^(24,27-34)^, Purtscher-like (PL) retinopathy, and autoimmune retinopathy^(35,36)^.

Lupus microangiopathy, the most common presentation, manifests as cotton wool spots, microaneurysms, hard exudatese, and intraretinal hemorrhages^(24,27-34)^. Visual acuity is good, unless there is macular involvement, and it generally has good visual prognosis^(37-40)^.

**Figure 1:**
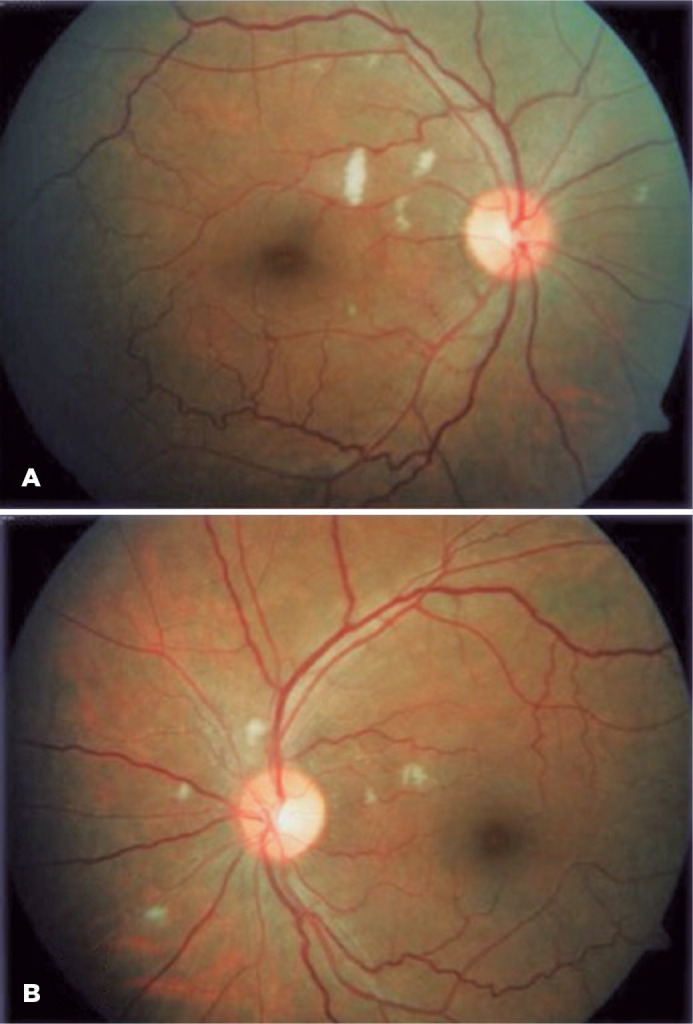
Lupus retinopathymicroangiopathycotton wool spots on both eyes.

Cotton wool spots are the clinical manifestation of microinfarctions of the retinal nerve fiber layer (Figure 1)^(27-34)^. They are caused by the interruption of the axoplasmic flow in retinal ganglion fibers. It is believed that this occurs due to ischemic retinal vasculitis affecting primarily the retinal arterioles. Although other diseases such as systemic arterial hypertension and diabetes also present with cotton wool spots, the retinal arterioles in these cases are attenuated and may often become occluded, resulting in a more severe ischemia than in SLE^(24)^.

**Figure 2:**
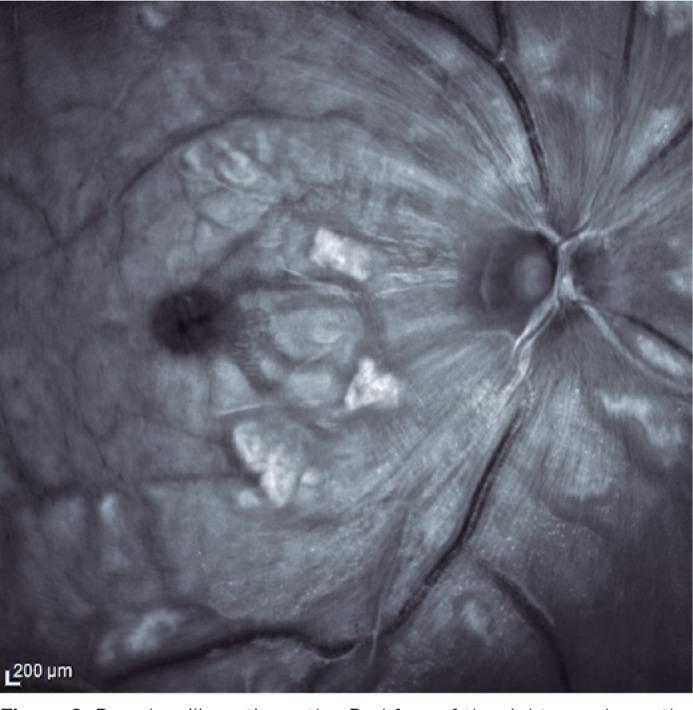
Purscher-like retinopathy. Red free of the right eye shows the presence of “fleckens,” well-defined whitish, polygonal areas.

Purtscher’s retinopathy was initially described as an ischemic retinopathy associated with trauma^(41-43)^. Other diseases, including SLE, can present with similar manifestations, and hence, they are termed as PL retinopathy (Figure 2). Its pathophysiology is believed to be due to the obstruction of the retinal microvasculature, leading to severe ischemia^(43-45)^. Fundoscopy shows areas of infarction of the inner layer of the retina and “fleckens,” well-defined whitish, polygonal areas that differ from cotton spots, because the latter are more superficial with a feathery appearance and blurred edges^(41-48)^. Hemorrhage and papilla edema may occur. PL retinopathy is generally associated with poor visual prognosis, even with early treatment, and may be the initial presentation or a sign of reactivation of SLE^(41-48)^.

**Figure 3:**
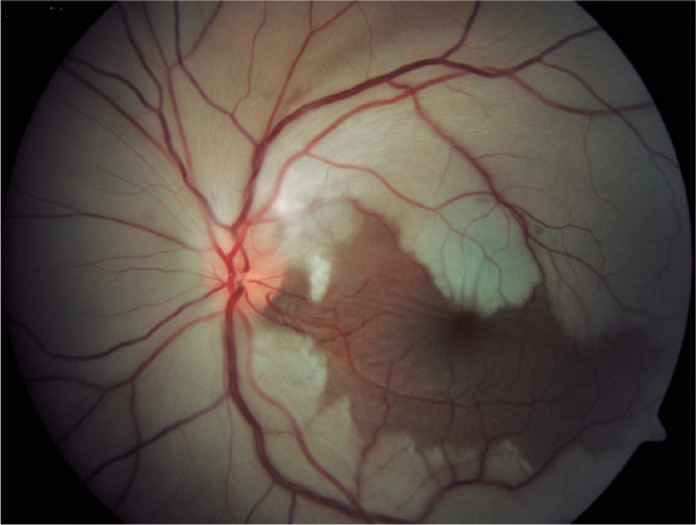
Lupus retinopathy. Occlusion of central retinal artery in the left eye leading to whitening of the retina with persistent cilioretinal artery, which maintains the normal aspect of the retina.

Retinal vascular occlusions occur when there are changes in blood flow in the retinal arteries or veins. They present as central venous occlusion of the retina, central retinal artery occlusion (Figure 3), or their branches^(24,27-34)^. Simultaneous venous and arterial occlusions in one or both eyes can occur. Arterial occlusive disease has been found to be more common than retinal vein occlusion^(24)^.

An association has been observed between antiphospholipid antibodies (aPL) and LR, implying that it is important to perform an ophthalmic examination in patients with SLE and aPL, as it is essential to examine the presence of aPL in patients with LR^(49-52)^. LR can resemble retinitis pigmentosa because a previous vascular occlusive disease results in retinal mottling and large clumps of pigment^(53)^.

**Figure 4:**
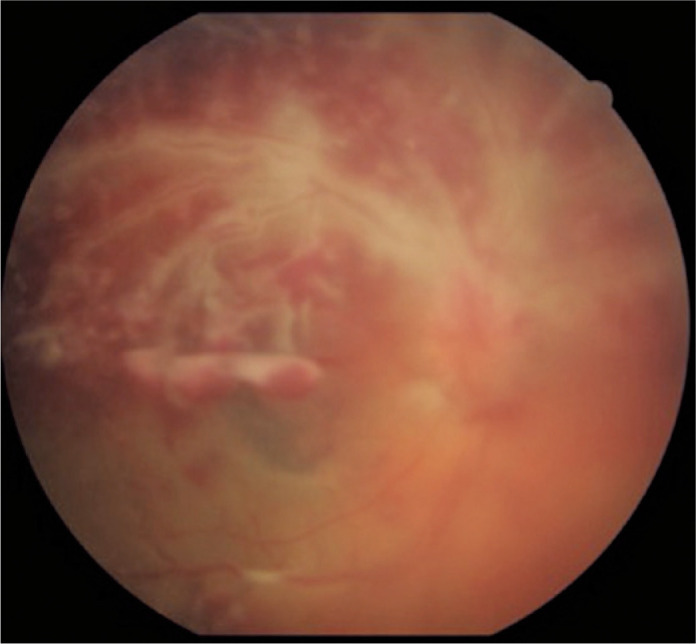
Lupus retinopathy presented as retinal vasculitis in the right eye. Optic disc edema, vascular sheath characterized by the adjacent whitening of vessels, especially in the superior arcade, and retinal hemorrhages are observed.

Retinal vasculitis is uncommon^(24,30,54)^, has an acute presentation^(30,54)^, may be localized or diffuse^(24,30,54)^, and is associated with poor visual outcome (Figure 4). It is characterized by diffuse arteriolar occlusion with extensive capillary nonperfusion, leading to retinal neovascularization.

Hypertensive retinopathy is generally associated with lupus nephritis, which causes a secondary hypertension. There may be arteriolar narrowing, pathologic arteriovenous crossing, cotton wool spots, hemorrhages, swollen papilla, and choroidal infarcts (Elschnig’s spots)^(27-34)^.

There have been few reports of autoimmune retinopathy, and it is believed that autoantibodies that affect photoreceptors can lead to the apoptosis of retinal cells and consequently cause visual dysfunction^(35,36)^.

The complications of LR are related to retinal ischemia with the formation of neovascularization, bleeding, vitreous opacity, and tractional retinal detachment. Other reports include serous retinal detachment associated with lupus choroidopathy and vascular tortuosity^(1,2,27-34)^.

Fluorescein angiography is useful for detecting vascular, macular, or optic nerve disease^(21,22)^ and the changes in eyes that appear clinically normal. These patients have no visual complaints. FA findings suggest an active disease or cerebral involvement; however, till date, there is no scientific evidence related to this theory^(21,22)^.

Optical coherence angiotomography (OCT-A) consists of angiotomographic evaluation of retinal vascularization based on the physical properties of interferometry^(20)^. Subclinical LR such as vasculitis and ischemia and can play a role in predicting severe systemic presentations of SLE. As it is a new examination in clinical practice, the literature supporting its practical utility in LR is limited^(20,55-57)^.

### Prevalence and risk factors of LR

Bergmeister et al. were the first to report LR in 1929. The LR lesions consisted of cotton wool spots and optic disc hyperemia. Before the pre-corticoid era, up to 50% of patients with SLE were reported to have retinal manifestations^(23)^.

Currently, due to the use of corticosteroids, immunosuppressants, and biological agents, the incidence of LR has dramatically decreased. The literature reports a varied prevalence of LR, ranging from 3% to 29%^(13)^. This prevalence gap can be justified by several factors such as the definition of LR used in different studies, the study design, the sample of patients, and geographical variations^(13,37-40)^. The most severe clinical LR presentations are rare and occur in <1% of patients^(58)^.

Since its first description in 1929, reports and case series have been published to gain a better understanding of LR and its role in the clinical spectrum of SLE. However, it was the emblematic study conducted by Sttaford-Brady et al. (1988) in Canada, which followed up a cohort of 550 patients with SLE over a period of 16 years, that set the basis for understanding LR. Sttaford-Brady et al. diagnosed 41 patients with LR and found microangiopathy as the primary clinical presentation, generally without visual acuity impairment. However, the cases of LR and low visual acuity, especially associated with venous or arterial vascular occlusions, tended to progress to irreversible visual loss. Sttaford-Brady et al. confirmed the presence of an association between LR and several factors, including active disease, decreased life expectancy, and imminent mortality in 10% of patients. That study demonstrated that retinal hemorrhage is the primary fundoscopic finding associated with increa sed mortality^(13)^.

Montehermoso et al. (1999) conducted a cross-sectional study in Spain evaluating 82 patients with SLE and identified 13 (15%) patients with LR^(49)^. They observed that vascular occlusions were more common than microangiopathy. Despite the severity of clinical presentation, their patients maintained good visual acuity, unlike those of previous studies^(13)^. A possible explanation was that those manifestations were associated with the presence of antiphospholipid syndrome, resulting in intravascular thrombosis rather than immune complex deposition^(49)^.

In their study, no association was detected between LR and disease activity, but an association was found between LR and aPL. However, the authors did not specify the index that was used to measure SLE disease activity^(49)^.

Ushiyama et al. (2000) conducted a cross-sectional study in Japan on 69 patients with SLE and found LR in approximately 10% of the patients. Microangiopathy was the primary manifestation of LR, and the patients had good visual acuity, corroborating previous studies^(13)^. There was also an association between LR and disease activity according to the SLEDAI^(39)^.

Gao et al. (2017) conducted a 10-year case-control study in China (2006-2016) and evaluated 5298 patients with SLE. They detected LR in 35 (0.66%) patients. This small number of patients with LR can be explained by the fact that no fundus examinations were performed in all patients. Instead, only patients with SLE with visual complaints underwent fundoscopy. Therefore, patients with asymptomatic LR were possibly undiagnosed, which could have been a bias. We presume that patients with SLE with visual complaints generally present with severe LR and poor visual prognosis. In their study, 80% of patients with LR had decreased visual acuity, 11.7% had visual field loss, and 5% had diplopia^(38)^.

Seth et al. (2018) conducted a cross-sectional study in India on 437 patients with SLE and identified 45 (10.7%) patients with LR. Lupus microangiopathy was the most common manifestation, and the patients had a high activity index, measured by SLEDAI, compared with the group without LR^(37)^.

Recently, Azevedo et al. (2019) performed a cross-sectional study in Brazil on 102 patients with SLE^(40)^. They eidentified 5 (4.9%) patients with LR, and till date, no studies on LR from Latin America have been found in major databases. As the clinical features of SLE are known to vary in different parts of the world, it is important to include data from Latin America^(40,59-61)^ (Table 1).

**Table 1:** Prevalence and incidence of lupus retinopathy in several studies

Author	Country	Year	Methodology	Patients, n	LR, n (%)
Sttaford-Brady et al. ^(13)^	Canada	1989	Cohort	550	41 (7%)
Montehermoso et al. ^(49)^	Spain	1999	Cross-sectional	82	13 (15%)
Ushiyama et al. ^(39)^	Japan	2000	Cross-sectional	69	7 (10%)
Gao et al. ^(38)^	China	2017	Case-control	5298	35 (0.6 %)
Seth et al. ^(37)^	Índia	2018	Cross-sectional	437	45(10%)
Azevedo et al. ^(40)^	Brazil	2019	Cross-sectional	102	5 (4.9%)

According to the abovementioned study, the prevalence of LR in Brazil is similar to that in other countries^(13,37-40)^. In that study, 77 outpatients and 25 hospitalized patients were examined, and of the five patients with LR, one was an outpatient. Despite a relatively high proportion of LR among hospitalized patients, only one patient was found to be symptomatic. Therefore, among outpatients, there was a 1.29% prevalence of LR, and among hospitalized patients, it was 16%. In the major LR studies, there is no information regarding the proportion of hospitalized patients versus outpatients. Hence, we believe that the ratio between outpatients and hospitalized patients could interfere with the overall prevalence of LR^(40)^.

Therefore, we believe that hospitalized patients may have undiagnosed LR as they have mild forms of LR and are not routinely examined by an ophthalmologist during hospitalization. The higher prevalence of LR among hospitalized patients may be related to poor medication compliance, resulting in disease activity and hospitalization. Further studies should specify the sample’s characteristics (especially hospitalized patients versus outpatients) to obtain more accurate data to compare the prevalence and clinical presentations of LR^(40)^.

Several studies have attempted to establish the relationship between clinical features or laboratory tests and LR, with conflicting results. Such findings may be justified by different methodologies, clinical-epidemiological characteristics of patients with SLE, and severity of the disease^(40)^. No association was found between sex^(13,38,40,49)^, age^(13,37,40,49)^, and disease duration^(38,40,49)^. Among the clinical manifestations, studies have described the association between LR and lupus nephritis^(37,39)^ and neuropsychiatric involvement (“Neuropsychiatric Systemic Lupus Erythematosus,” NPSLE)^(13,37,39)^. It is believed that NPSLE could be related to LR due to the similar pathophysiological mechanism, which involves autoantibodies and immune complex deposition. Therefore, fundoscopy may be a useful, noninvasive tool for the indirect assessment of CNS microvascular damage in patients with SLE^(13)^. Seth et al. described an association between LR, autoimmune hemolytic anemia, and serositis. In contrast, Gao et al. found no association between LR and malar rash, photosensitivity, and arthritis^(38)^.

In addition, no association was reported between erythrocyte sedimentation rate (ESR)^(38,40)^, C-reactive protein (CRP)^(38,40)^, platelet count^(38,40)^, and C3 and C4^(37)^ levels. Gao et al.^(38)^ described an association between leukopenia and LR, whereas other studies did not corroborate this finding^(37,39)^.

Among autoantibodies, anti-DNA^(37,38,40,49)^, anti-LA^(37)^, and anti-RNP were not associated with LR^(37,38)^. Antiphospholipid antibodies may play a role because it is possible that the formation of microthrombi in the retinal microvasculature causes retinal vascular occlusions^(39,49)^. Anti-SM is a specific autoantibody of SLE, and Seth et al. ^(37)^ described an association with LR; however, this finding was not described by Gao et al. ^(38)^

It is important to mention that Gao et al. described an inverse relationship between anti-Ro and LR, and hence this autoantibody would be a protective factor for LR, which was not mentioned in other studies. A possible explanation for the controversial results of Gao et al. is the study methodology, because LR was retrospectively evaluated and only in symptomatic patients. Table 2 summarizes the major association between clinical findings or laboratory tests and LR^(38)^.

**Table 2:** Major association between clinical and laboratory lupus retinopathy

	Sex	Age	SLE- duration	SLEDAI	PCR	ESR	Anti-DNA	aCL	Renal disease	Neuro-SLE
Sttaford-Brady et al.^(13)^	-	-	-	N	N	N	N	N	-	+
Ushiyama et al.^(39)^	-	-	-	+	N	N	N	+	+	+
Gao et al.^(38)^	-	-	-	+	-	-	-	-	-	+
Seth et al.^(37)^	-	-	-	+	N	N	-	-	+	+
Azevedo et al.^(40)^	-	-	-	+	-	-	-	N	N	N

Systemic lupus erythematosus disease activity index (SLEDAI); C-reactive protein (CRP); Erythrocyte sedimentation rate (ESR); Double-stranded deoxyribonucleic acid antibody (anti-DNA); anticardiolipin antibody (Acl); Neuropsychiatric systemic lupus erythematosus (Neuro-SLE). Statistically significant association with LR (+); Statistically nonsignificant association with LR (-); Not validated (N).

In conclusion, in Brazil, there are multiple ethnicities and intense miscegenation, unlike other countries. Although Azevedo et al. did not find an association between LR and ethnicity^(40)^, it is not known whether miscegenation influenced this result Therefore, further research is required to answer these questions.

Currently, there are no protocols recommending ophthalmic examination in patients with SLE. Considering the relationship between LR and SLE mortality, fundoscopy plays a vital role in the follow-up of these patients. We believe that fundoscopy should be conducted at the time of diagnosis, in patients with complaints of acute visual impairment, in those with a high SLEDAI score, without treatment, hospitalized patients, or those with aPL. For asymptomatic patients, we suggest an annual ophthalmological assessment to evaluate the side effects of medications such as cataract and glaucoma related to corticosteroids and hydroxychloroquine maculopathy. Fluorescein angiography and, more recently, OCT-A are complementary methods to evaluate LR, especially in patients with SLE without fundus changes but with risk factors for LR, to detect subclinical forms of LR.
